# MRI evidence of structural changes in the sacroiliac joints of patients with non-radiographic axial spondyloarthritis even in the absence of MRI inflammation

**DOI:** 10.1186/s13075-017-1342-9

**Published:** 2017-06-06

**Authors:** Walter P. Maksymowych, Stephanie Wichuk, Maxime Dougados, Heather Jones, Annette Szumski, Jack F. Bukowski, Lisa Marshall, Robert G. Lambert

**Affiliations:** 1 Department of Medicine, University of Alberta, 568 Heritage Medical Research Building, Edmonton, AB T6G 2S2 Canada; 2 Paris Descartes University; Department of Rheumatology – Hôpital Cochin, Assistance Publique – Hôpitaux de Paris; INSERM (U1153): Clinical Epidemiology and Biostatistics, PRES Sorbonne Paris-Cité, Paris, France; 3 Pfizer, Collegeville, PA USA; 4 inVentiv Health, Princeton, NJ USA; 5 Department of Radiology and Diagnostic Imaging, University of Alberta, Edmonton, AB Canada

**Keywords:** Non-radiographic axial spondyloarthritis, Magnetic resonance imaging, Sacroiliac joint, Structural lesion

## Abstract

**Background:**

Studies have shown that structural lesions may be present in patients with non-radiographic axial spondyloarthritis (nr-axSpA). However, the relevance of structural lesions in these patients is unclear, particularly without signs of inflammation on magnetic resonance imaging (MRI). We assessed the presence of structural lesions at baseline on MRI in the sacroiliac joints (SIJ) of patients with nr-axSpA with and without SIJ inflammation on MRI.

**Methods:**

Bone marrow edema (BME) was assessed on short tau inversion recovery (STIR) scans from 185 patients with nr-axSpA, by two independent readers at baseline using the Spondyloarthritis Research Consortium of Canada (SPARCC) score. Structural lesions were evaluated on T1 weighted spin echo scans, with readers blinded to STIR scans, using the SPARCC MRI SIJ structural score. Disease characteristics and structural lesions were compared in patients with SIJ BME (score ≥2) and without SIJ BME (score <2).

**Results:**

Both SIJ BME and structural lesions scores were available for 183 patients; 128/183 (69.9%) patients had SIJ BME scores ≥2 and 55/183 (30.1%) had scores <2. Frequencies of MRI structural lesions in patients with vs without SIJ BME were: erosions (45.3% vs 10.9%, *P <* 0.001), backfill (20.3% vs 0%, *P* < 0.001), fat metaplasia (10.9% vs 1.8%, *P =* 0.04), and ankylosis (2.3% vs 1.8%, *P =* ns). Significantly more patients with both SIJ BME and structural lesions were male and/or HLA-B27 positive than patients with only SIJ BME. Mean (SD) spinal scores (23 discovertebral units) were significantly higher in patients with SIJ structural lesions than without: 6.5 (11.5) vs 3.3 (5.1), respectively, *P* = 0.01.

**Conclusions:**

In patients with nr-axSpA, SIJ structural lesions, particularly erosions, may be present on MRI when radiographs are normal or inconclusive, even in patients negative for MRI SIJ inflammation. They may reflect more severe disease with greater spinal inflammation.

**Trial Registration:**

ClinicalTrials.gov, NCT01258738. Registered on 9 December 2010.

**Electronic supplementary material:**

The online version of this article (doi:10.1186/s13075-017-1342-9) contains supplementary material, which is available to authorized users.

## Background

Treatment of axial spondyloarthritis (axSpA) is increasingly aimed at intervention early in the disease course before radiographic sacroiliitis has appeared and when response to treatment is greatest. Thus, magnetic resonance imaging (MRI) is an important tool for the diagnosis of SpA because it can identify inflammation prior to the detection of radiographic changes [1–5].

The Assessment of SpondyloArthritis international Society (ASAS) classification system for axSpA is based on whether patients meet clinical criteria or imaging criteria [6–8]. Patients who meet the ASAS criteria for axSpA and do not meet the modified New York criteria for radiographic sacroiliitis are considered to have non-radiographic axSpA (nr-axSpA). According to the ASAS classification criteria, bone marrow edema (BME)/osteitis on MRI is a requirement for patients with nr-axSpA who meet the criteria via the MRI arm [8]. However, this may also occur nonspecifically, and some patients with nr-axSpA do not have MRI evidence of inflammation [1, 2].

Recent studies have described several structural lesions on MRI in the sacroiliac joints (SIJ) [9–11]. Erosion is considered present when there is a breach in the cortical bone of either the iliac or sacral bones, which appears dark on both T1 weighted spin echo (T1WSE) and short tau inversion recovery (STIR) sequences, together with loss of the bright signal from adjacent marrow matrix on T1WSE MRI [12]. The T1WSE MRI sequence may also reveal a lesion in bone marrow adjacent to subchondral bone characterized by a homogeneous increase in marrow signal, indicative of accumulation of lipid, and a distinct border [10]. The histopathologic nature of this lesion remains unclear; while it may reflect adipose tissue, it may also reflect lipid accumulation in various cell types, which can occur during the course of differentiation into different phenotypes. Because it has now been shown that this lesion indicates tissue transformation following resolution of BME, it has been termed fat metaplasia [9, 13]. Recent work has shown that similar tissue develops at sites of erosion following resolution of inflammation; this has been termed “backfill” [9]. Standardized definitions for these diverse lesions have recently been developed and validated together with a semi-quantitative scoring method that assesses each individual lesion [10].

Although limited studies have demonstrated that structural lesions may already be evident in non-radiographic SpA defined according to physician expert opinion [1, 2], the majority of these patients had concomitant BME. Thus, it is unclear to what degree these diverse lesions are relevant in patients classified according to ASAS criteria, particularly in the absence of MRI inflammation, such as patients who meet the clinical but not the imaging arm of the criteria. We therefore aimed to assess the relative frequency of structural and inflammatory lesions as seen on MRI in the SIJ of patients with nr-axSpA. We also compared the disease characteristics and severity in patients with and without MRI SIJ structural lesions, anticipating that these lesions might reflect a more severe phenotype of disease.

## Methods

Details of the EMBARK study (ClinicalTrials.gov identifier: NCT01258738), a phase IIIb, 104-week clinical study, have been published previously [14, 15]. Briefly, patients had axSpA per the ASAS classification criteria, were aged 18–49 years, had symptoms for >3 months and <5 years, Bath Ankylosing Spondylitis Disease Activity Index (BASDAI) score ≥4, and had an inadequate response to ≥2 nonsteroidal anti-inflammatory drugs (NSAIDs). Patients were excluded if they met the modified New York criteria for radiographic axSpA or if they had ever received a tumor necrosis factor (TNF) inhibitor for a condition other than inflammatory bowel disease. Patients met either the ASAS imaging criteria (sacroiliitis on imaging and ≥1 SpA feature) or the clinical criteria (human leukocyte antigen (HLA)-B27 positive and ≥2 other SpA features). Patients were randomized to double-blind etanercept 50 mg/week or placebo for 12 weeks, and then all patients were switched to etanercept for the remainder of the study [14]. For this report, both treatment arms were pooled and only baseline data are presented.

The study was conducted in accordance with the International Conference on Harmonisation guidelines for Good Clinical Practice and the ethical principles of the Declaration of Helsinki. Written informed consent was obtained from all patients prior to enrollment. The institutional review board or independent ethics committee at each participating center reviewed and approved all consent forms and the study protocol (see “Acknowledgments” for details).

This was a post-hoc cross-sectional analysis of patients with baseline lesion scores. The primary analysis was assessment of the presence of structural lesions on MRI in patients with SIJ BME (score ≥2) versus without SIJ BME (score <2). Sensitivity analysis was performed by evaluating the presence of structural lesions in patients who were ASAS MRI positive versus negative.

### Evaluation of BME of the SIJ and spine

Two independent central readers, blinded to treatment and patient characteristics, assessed STIR scans of the SIJ and spine for BME using the Spondyloarthritis Research Consortium of Canada (SPARCC) scores [16–18]. For all assessments, the mean score of the two readers was used [14]. An adjudicator reviewed MRI scans with pre-specified reader discrepancies, and in these cases, the mean of all three readers’ scores was used. The pre-specified reader discrepancies are provided in Additional file 1: Table S1. Spinal BME was evaluated using the 23-discovertebral unit (DVU) score, which ranges from 0 to 414. This scoring system assesses all 23 vertebral units of the spine from vertebrae C2/3 to L5/S1 [18]. A score ≥2 for SIJ BME was considered positive MRI evidence of inflammation, as reported previously [19]. This cutoff has been validated in the literature [20].

### Evaluation of structural lesions

**Fig. 1:**
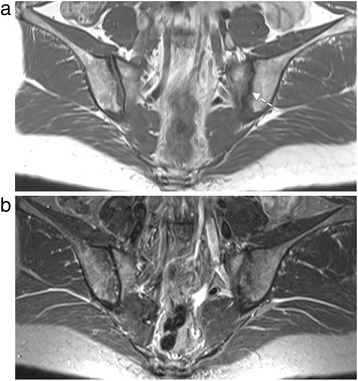
Erosion of the left iliac bone on T1 weighted spin echo magnetic resonance imaging (MRI) (**a**) and in the absence of inflammation on short tau inversion recovery MRI (**b**)

Two different readers (who developed the SPARCC scoring method), scored structural lesions using the SPARCC MRI SIJ structural score (SSS), which assesses fat metaplasia, erosion, backfill, and ankylosis on T1WSE MRI [10]. The readers were independent and blinded to patient characteristics, treatment, and the corresponding STIR scan. Figure 1 shows an example of erosion at baseline on T1WSE MRI in a case that was negative on STIR MRI.

The two readers scored the presence of lesions dichotomously (yes/no) in SIJ quadrants for erosion and fat metaplasia and in SIJ halves for backfill and ankylosis using a data entry system based on a diagram of the SIJ [10]. Five consecutive semi-coronal slices were evaluated beginning with the transitional slice and scrolling anteriorly. The transitional slice was defined as the first cartilaginous slice with a visible section of the ligamentous joint when assessed anteriorly to posteriorly [10]. Erosion and fat metaplasia each received a score of 0–8 per slice for five slices, for a total score of 0–40. Backfill and ankylosis each received a score of 0–4 per slice for five slices, for a total score of 0–20. A SPARCC SSS >0 was required from both readers for identification of a structural lesion of erosion, backfill, fat metaplasia, or ankylosis.

### Structural lesions seen on T1WSE MRI: standardized definitions

This study used standardized definitions of structural lesions of the SIJ on MRI, which were developed by the Canada-Denmark MRI Working Group and then extended in a subsequent report to include backfill [9, 10, 12]. These definitions are provided below. See Additional file 2 for images of the lesion types on T1WSE MRI.

- Erosion: on T1WSE is the full-thickness loss of the dark appearance of iliac or sacral cortical bone at its anticipated location as well as loss of the normal bright appearance of adjacent bone marrow.
- Backfill: on T1WSE is the complete loss of iliac or sacral cortical bone at its anticipated location as well as increased signal clearly demarcated from adjacent normal marrow by dark signal with irregular contour reflecting sclerosis at the border of the eroded bone.
- Fat metaplasia: increased signal on T1WSE sequences. To be scored in the SPARCC SSS method, there must be homogeneous signal across the lesion, and it must extend >1 cm in depth from the joint surface.
- Ankylosis: bone marrow signal on T1WSE between the sacral and iliac bone marrow with a full-thickness loss of the dark appearance of the iliac and sacral cortical bone.

### Statistical analysis

Proportions of patients with lesions scored >0 (i.e. with any lesions or specific lesions) were determined according to the presence or absence of SIJ BME (primary strata) and according to ASAS MRI positive and negative status (secondary strata); differences in proportions were tested using Cochran-Mantel-Haenszel (CMH) tests. The presence of SIJ BME and structural lesions was evaluated according to the radiographic grade of sacroiliitis using CMH tests. Differences in demographics and baseline disease characteristics, including 23-DVU spinal score, were analyzed by presence or absence of any SSS lesions (>0 or =0) overall and separately by presence or absence of SIJ BME (≥2 or <2). Each type of structural lesion was evaluated in this manner as well. These analyses were performed using analysis of variance, the chi-square test, or the Wilcoxon-Mann-Whitney test.

Cumulative probability plots were derived to compare the SSS lesion scores of specific structural lesions according to the presence or absence of SIJ BME. These data were also analyzed using simple descriptive statistics with one-way analysis of variance and the Wilcoxon-Mann-Whitney test to test for differences in SIJ BME subgroups. Inter-observer intra-class correlation coefficients (ICC) were determined for structural lesion scores to evaluate scoring consistency between the two MRI readers. Additionally, the proportion of patients with individual lesion scores >0 was compared between two different definitions: scores from both readers were >0, and the score from at least one reader was >0. Spearman correlation was tested to analyze the relationship between continuous spinal inflammation, i.e. the 23-DVU spinal score, and continuous patient characteristics, i.e. age and symptom duration, and between continuous spinal inflammation and continuous structural lesions. Multivariate stepwise regression analysis was used to determine whether age, gender, symptom duration, presence/absence of SIJ BME, or presence of specific structural lesions was significantly associated with the 23-DVU spinal score.

## Results

### Patients

MRI scans at baseline were available for 185 patients. The mean (standard deviation (SD)) age was 32.0 (7.8) years; 60.5% were male, and the mean (SD) duration of disease symptoms was 2.4 (1.8) years. A total of 133/182 (71.9%) patients were HLA-B27 positive and 152/185 (82.2%) met the ASAS MRI imaging criteria.

**Fig. 2:**
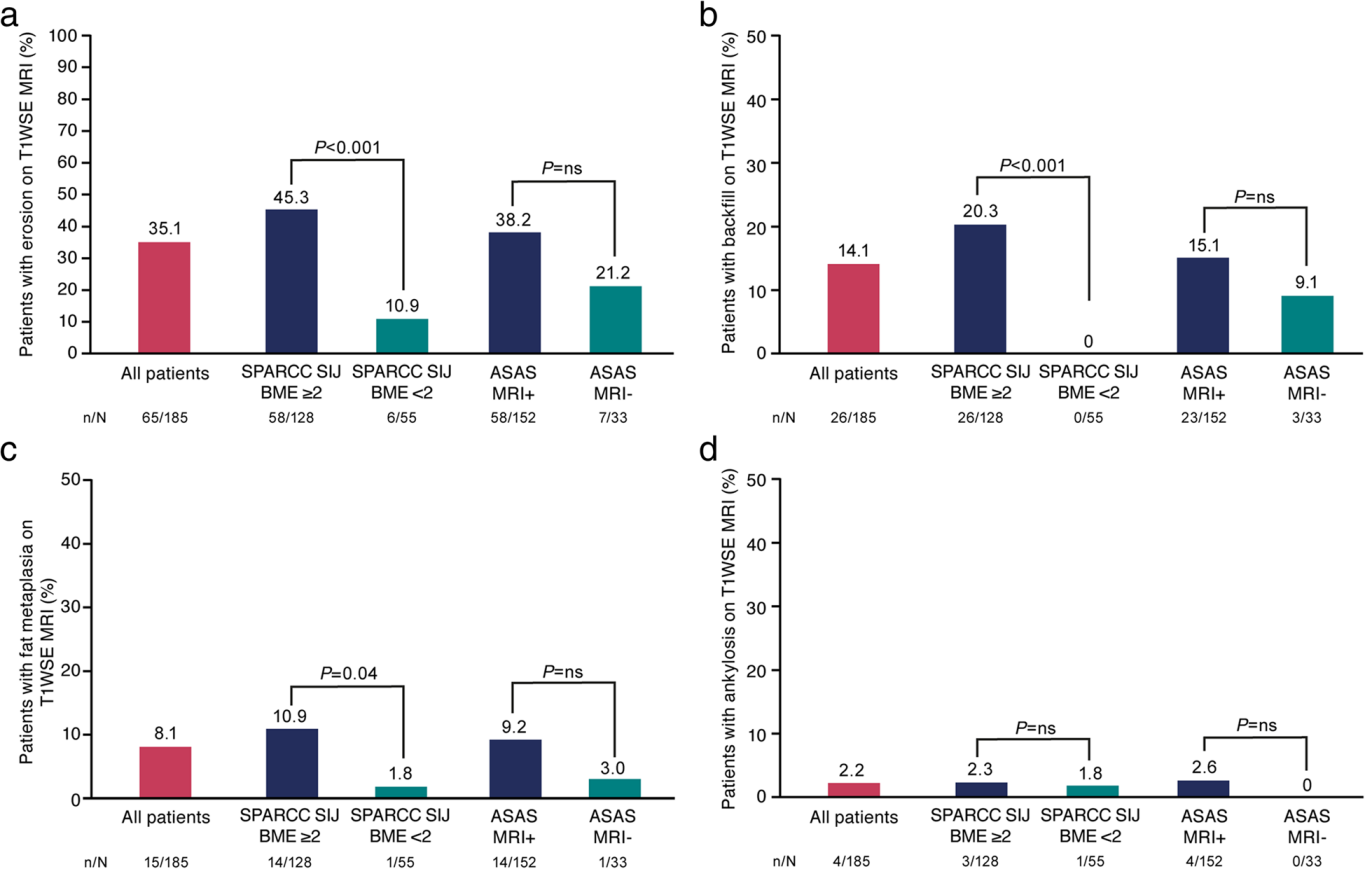
Proportion of patients with erosion (**a**), backfill (**b**), fat metaplasia (**c**), and ankylosis (**d**) on T1 weighted spin echo (*T1WSE*) magnetic resonance imaging (*MRI*). *ns* nonsignificant, *SPARCC* Spondyloarthritis Research Consortium of Canada, *SIJ*, sacroiliac joint, *BME* bone marrow edema, *ASAS* Assessment of SpondyloArthritis international Society

A total of 77/185 (41.6%) patients had ≥1 structural lesion on MRI comprising erosion (65/185, 35.1%), backfill (26/185, 14.1%), fat metaplasia (15/185, 8.1%), and ankylosis (4/185, 2.2%). SIJ BME data were available for 183 patients; 128/183 (69.9%) patients had SIJ BME and 55/183 (30.1%) did not. Of the patients with SIJ BME, 69/128 (53.9%) had a structural lesion on MRI, compared with 7/55 (12.7%) patients without SIJ BME (*P* < 0.001). Relative frequencies of MRI structural lesions in patients with versus without SIJ BME were: erosion (58/128, 45.3% vs 6/55, 10.9%; *P* < 0.001), backfill (26/128, 20.3% vs 0/55, 0%; *P* < 0.001), fat metaplasia (14/128, 10.9% vs 1/55, 1.8%; *P* = 0.04), and ankylosis (3/128, 2.3% vs 1/55, 1.8%; *P* = nonsignificant (ns)) (Fig. 2).

Of the patients who were ASAS MRI positive, 69/152 (45.4%) had a structural lesion on MRI, compared with 8/33 (24.2%) who were ASAS MRI negative (*P* = 0.03). For ASAS MRI positive versus negative patients, relative frequencies of structural lesions were: erosion (58/152, 38.2% vs 7/33, 21.2%; *P* = ns), backfill (23/152, 15.1% vs 3/33, 9.1%; *P* = ns), fat metaplasia (14/152, 9.2% vs 1/33, 3.0%; *P* = ns), and ankylosis (4/152, 2.6% vs 0/33, 0%; *P* = ns) (Fig. 2).

A comparison of the demographics and baseline disease characteristics between patients with the presence (SSS >0) or absence (SSS = 0) of any structural lesion according to those with and without SIJ BME is provided in Table 1. The percentage of male patients was significantly higher in the group with both SIJ BME and structural lesions (82.6% male) than in the group with only SIJ BME (47.5%, *P* < 0.001). Additionally, age was significantly lower in the group with both SIJ BME and structural lesions (30.3 years) compared to the group with only SIJ BME (33.5 years, *P* = 0.01). Symptom duration was similar across the groups. More patients were HLA-B27-positive who had both SIJ BME and structural lesions (87.0%) than those with only SIJ BME (62.7%, *P* = 0.002). Mean BASDAI and mean BASFI were significantly higher in the group with SIJ BME and no structural lesions (6.4 cm and 4.6 cm, respectively) than in the group with both SIJ BME and structural lesions (5.7 cm and 3.7 cm, *P* = 0.03 and 0.04, respectively). For the patients without SIJ BME, the small sample size of only seven patients in the SSS >0 category precluded interpretation of this subgroup.

**Table 1:** Demographics and baseline disease characteristics according to the presence or absence of SIJ inflammation and structural lesions

	SPARCC SIJ BME ≥2 (*N* = 128)			SPARCC SIJ BME <2 (*N* = 55)			Total (*N* = 183)		
	SSS >0 *n* = 69	SSS = 0 *n* = 59	*P* value	SSS >0 *n* = 7	SSS = 0 *n* = 48	*P* value	SSS >0 *n* = 76	SSS = 0 *n* = 107	*P* value
Age, years*	30.3 (6.8)	33.5 (7.7)	0.01	33.9 (11.9)	32.6 (8.5)	0.73	30.6 (7.3)	33.1 (8.0)	0.04
Male, *n* (%)^†^	57 (82.6)	28 (47.5)	<0.001	3 (42.9)	22 (45.8)	1.0	60 (79.0)	50 (46.7)	<0.001
BMI, kg/m^2^*	24.5 (3.6)	25.2 (4.6)	0.34	25.3 (5.6)	25.4 (5.2)	0.96	24.6 (3.8)	25.3 (4.9)	0.29
Symptom duration, years*	2.5 (2.2)	2.7 (1.4)	0.47	2.6 (1.5)	1.9 (1.4)	0.22	2.5 (2.2)	2.4 (1.4)	0.64
HLA-B27 positive, *n* (%)^‡^	60 (87.0)	37 (62.7)	0.002	6 (85.7)	29 (60.4)	0.40	66 (86.8)	66 (61.7)	<0.001
Anterior uveitis, *n* (%)^‡^	7 (10.1)	6 (10.2)	1.0	0	1 (2.1)	1.0	7 (9.2)	7 (6.5)	0.58
IBD, *n* (%)^‡^	0	0	1.0	0	1 (2.1)	1.0	0	1 (0.9)	1.0
PsO, *n* (%)^‡^	8 (11.6)	10 (17.0)	0.45	0	5 (10.4)	1.0	8 (10.5)	15 (14.0)	0.51
PsO family history, *n* (%)^‡^	4 (5.8)	1 (1.7)	0.38	0	3 (6.3)	1.0	4 (5.3)	4 (3.7)	0.72
ASDAS-CRP	3.1 (1.1)	3.1 (0.9)	0.98	2.3 (1.2)	2.8 (0.8)	0.11	3.0 (1.1)	3.0 (0.9)	0.85
CRP, mg/l	7.7 (9.6)	7.6 (14.7)	0.96	5.3 (9.3)	5.0 (7.0)	0.93	7.5 (9.6)	6.4 (11.9)	0.53
BASDAI, 0–10	5.7 (2.0)	6.4 (1.6)	0.03	4.5 (2.3)	6.0 (1.6)	0.03	5.6 (2.0)	6.2 (1.6)	0.02
BASFI, 0–10 cm VAS	3.7 (2.5)	4.6 (2.6)	0.04	3.0 (1.4)	3.9 (2.2)	0.30	3.6 (2.5)	4.3 (2.4)	0.07
BASMI, 0–10	1.0 (1.2)	1.4 (1.5)	0.14	1.2 (0.9)	1.4 (1.4)	0.72	1.1 (1.2)	1.4 (1.4)	0.10
SPARCC spine score, 23-DVU, 0–414	6.9 (11.9)	4.0 (5.8)	0.10	2.5 (3.9)	2.5 (3.9)	0.99	6.5 (11.5)	3.3 (5.1)	0.01
SPARCC spine score, 23-DVU 0–414, median^§^	2.0	1.5	0.39	1.0	0.8	0.85	2.0	1.5	0.12

Values are mean (standard deviation) unless stated otherwise. *P* values are from one-way analysis of variance (ANOVA) with treatment as factor, unless stated otherwise. *ASDAS-CRP* Ankylosing Spondylitis Disease Activity Score based on C-reactive protein, *BASDAI* Bath Ankylosing Spondylitis Disease Activity Index, *BASFI* Bath Ankylosing Spondylitis Functional Index, *BASMI* Bath Ankylosing Spondylitis Metrology Index, *BME* bone marrow edema, *BMI* body mass index, *CRP* C-reactive protein, *DVU* discovertebral units, *HLA* human leukocyte antigen, *IBD* inflammatory bowel disease, *PsO* psoriasis, *SIJ* sacroiliac joint, *SSS* SIJ structural score, *VAS* visual analog scale

*P values calculated using one-way ANOVA comparing means between groups

^†^ *P* values from the chi-square test

^‡^ *P* values from Fisher’s exact test (two-tailed)

^§^ *P* value from the Wilcoxon-Mann-Whitney test

When characterized according to the presence or absence of structural lesions in the SIJ, mean (SD) 23-DVU spinal scores were higher overall in those patients with structural lesions in the SIJ: 6.5 (11.5) vs 3.3 (5.1), respectively, *P* = 0.01. Similar differences in demographic and baseline disease characteristics were also observed when comparisons were made according to the presence or absence of individual structural lesions, with more male patients, higher B27 positivity, and greater spinal inflammation, especially in patients with erosion or backfill (Table 2).

**Table 2:** Demographics and baseline disease characteristics according to baseline SIJ inflammation and structural lesions

	SPARCC SIJ BME	Erosion		Backfill		Fat metaplasia		Ankylosis	
	≥2 *n* = 128	>0 *n* = 58	0 *n* = 70	>0 *n* = 26	0 *n* = 102	>0 *n* = 14	0 *n* = 114	>0 *n* = 3	0 *n* = 125
	<2 *n* = 55	>0 *n* = 6	0 *n* = 49	>0 *n* = 0	0 *n* = 55	>0 *n* = 1	0 *n* = 54	>0 *n* = 1	0 *n* = 54
Age, years	≥2	30.0 (6.1)	33.2 (8.0)	28.9 (6.9)	32.5 (7.3)	32.3 (7.6)	31.7 (7.3)	26.3 (1.2)	31.9 (7.4)
	<2	35.3 (12.4)	32.4 (8.4)	─	32.7 (8.8)	40.0	32.6 (8.9)	25.0	32.9 (8.9)
Male, *n* (%)	≥2	51 (87.9)	34 (48.6)	21 (80.8)	64 (62.8)	12 (85.7)	73 (64.0)	3 (100)	82 (65.6)
	<2	3 (50.0)	22 (44.9)	─	25 (45.5)	1 (100)	24 (44.4)	0	25 (46.3)
BMI, kg/m^2^	≥2	24.6 (3.5)	25.1 (4.5)	24.7 (3.8)	24.9 (4.2)	25.1 (3.1)	24.8 (4.2)	25.9 (4.9)	24.8 (4.1)
	<2	26.7 (4.8)	25.3 (5.3)	─	25.4 (5.2)	30.1	25.4 (5.3)	17.3	25.6 (5.2)
Symptom duration, years	≥2	2.5 (2.3)	2.7 (1.5)	3.0 (2.9)	2.5 (1.5)	3.2 (1.7)	2.5 (1.9)	4.0 (1.2)	2.5 (1.9)
	<2	2.4 (1.5)	2.0 (1.4)	─	2.0 (1.4)	0.3	2.0 (1.4)	4.0	2.0 (1.4)
HLA-B27 positive, *n* (%)	≥2	50 (86.2)	47 (67.1)	25 (96.2)	72 (70.6)	12 (85.7)	85 (74.6)	3 (100)	94 (75.2)
	<2	5 (83.3)	30 (61.2)	─	35 (63.6)	1 (100)	34 (63.0)	1 (100)	34 (63.0)
Anterior uveitis, *n* (%)	≥2	5 (8.6)	8 (11.4)	1 (3.9)	12 (11.8)	2 (14.3)	11 (9.7)	1 (33.3)	12 (9.6)
	<2	0	1 (2.0)	─	1 (1.8)	0	1 (1.9)	0	1 (1.9)
IBD, *n* (%)	≥2	0	0	0	0	0	0	0	0
	<2	0	1 (2.0)	─	1 (1.8)	0	1 (1.9)	0	1 (1.9)
PsO, *n* (%)	≥2	6 (10.3)	12 (17.1)	2 (7.7)	16 (15.7)	3 (21.4)	15 (13.2)	0	18 (14.4)
	<2	0	5 (10.2)	─	5 (9.1)	0	3 (5.6)	0	5 (9.3)
PsO family history, *n* (%)	≥2	4 (6.9)	1 (1.4)	0	5 (4.9)	1 (7.1)	4 (3.5)	0	5 (4.0)
	<2	0	3 (6.1)	─	3 (5.5)	0	3 (5.6)	0	3 (5.6)
ASDAS-CRP	≥2	3.0 (1.1)	3.1 (0.9)	3.2 (1.1)	3.0 (1.0)	2.7 (0.7)	3.1 (1.0)	3.1 (0.1)	3.1 (1.0)
	<2	2.4 (1.3)	2.8 (0.8)	─	2.8 (0.9)	0.7	2.8 (0.8)	1.8	2.8 (0.9)
CRP, mg/l	≥2	7.6 (9.7)	7.7 (13.9)	8.6 (9.5)	7.4 (12.8)	4.0 (4.1)	8.1 (12.8)	2.8 (2.2)	7.7 (12.3)
	<2	5.8 (10.0)	4.9 (7.0)	─	5.0 (7.2)	1.3	5.1 (7.3)	2.2	5.1 (7.3)
BASDAI, 0–10	≥2	5.6 (2.0)	6.4 (1.7)	5.9 (2.3)	6.1 (1.7)	5.4 (1.9)	6.1 (1.8)	6.8 (1.2)	6.0 (1.9)
	<2	4.7 (2.5)	5.9 (1.6)	─	5.8 (1.7)	0.4	5.9 (1.6)	3.5	5.8 (1.7)
BASFI, 0–10 cm VAS	≥2	3.6 (2.6)	4.5 (2.5)	3.7 (2.8)	4.2 (2.5)	3.6 (2.0)	4.2 (2.6)	4.5 (0.7)	4.1 (2.6)
	<2	3.0 (1.6)	3.9 (2.2)	─	3.8 (2.1)	0.5	3.8 (2.1)	2.9	3.8 (2.2)
BASMI, 0–10	≥2	1.1 (1.3)	1.3 (1.4)	1.1 (1.5)	1.2 (1.3)	0.9 (1.1)	1.3 (1.4)	1.3 (2.2)	1.2 (1.3)
	<2	1.2 (1.0)	1.4 (1.3)	─	1.3 (1.3)	0	1.4 (1.3)	1.3	1.3 (1.3)
SPARCC spine score, 23-DVU, 0–414	≥2	7.5 (12.8)	4.0 (5.7)	9.1 (16.1)	4.6 (7.1)	5.6 (7.7)	5.5 (9.9)	11.7 (14.9)	5.4 (9.6)
	<2	2.8 (4.2)	2.5 (3.9)	─	2.5 (3.9)	3.5	2.5 (3.9)	1.0	2.5 (3.9)

Values are mean (standard deviation) unless stated otherwise. *ASDAS-CRP* Ankylosing Spondylitis Disease Activity Score based on C-reactive protein, *BASDAI* Bath Ankylosing Spondylitis Disease Activity Index, *BASFI* Bath Ankylosing Spondylitis Functional Index, *BASMI* Bath Ankylosing Spondylitis Metrology Index, *BME* bone marrow edema, *BMI* body mass index, *CRP* C-reactive protein, *DVU* discovertebral units, *HLA* human leukocyte antigen, *IBD* inflammatory bowel disease, *PsO* psoriasis, *SIJ* sacroiliac joint, *SPARCC* Spondyloarthritis Research Consortium of Canada, *VAS* visual analog scale

**Fig. 3:**
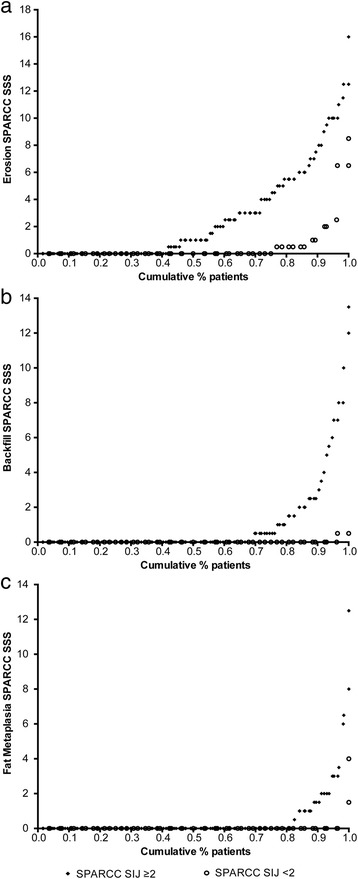
Cumulative probability plots for structural lesions according to Spondyloarthritis Research Consortium of Canada (*SPARCC*) sacroiliac joint (*SIJ*) score (≥2 vs <2): erosion (**a**), backfill (**b**), and fat metaplasia (**c**)

The cumulative probability plots demonstrate visually that the structural lesions of erosion, backfill, and fat metaplasia occurred to a greater extent in patients with than without SIJ BME (Fig. 3), which is confirmed by the significant differences in these SIJ BME subgroups in the Wilcoxon-Mann-Whitney tests (see Additional file 1: Table S2). For ankylosis, there was little difference between the patients with and without SIJ BME. The mean (SD) and median (Q1, Q3) SPARCC SSS values for the individual structural lesions according to the presence or absence of SIJ BME are provided in Additional file 1: Table S2.

### MRI structural lesions and SIJ BME according to radiographic grade

An analysis of the presence of SIJ BME and structural lesions according to radiographic grade did not identify a linear relationship between these measurements. However, the frequency of MRI structural lesions was greatest in those patients with radiographic unilateral grade 1 and contralateral grade 2 changes in the SIJ (Table 3).

**Table 3:** MRI inflammatory and structural lesions according to radiographic grading of sacroiliitis

Radiographic grade	MRI inflammation	MRI structural lesion				
	SPARCC SIJ BME ≥ 2	SSS > 0	Erosion > 0	Fat Metaplasia > 0	Backfill > 0	Ankylosis > 0
Bilateral grade 0	23/37 (62.2)	11/38 (28.9)	10/38 (26.3)	2/38 (5.3)	3/38 (7.9)	0/38 (0)
Unilateral grade 1	25/40 (62.5)	10/41 (24.4)	10/41 (24.4)	2/41 (4.9)	2/41 (4.9)	0/41 (0)
Unilateral grade 2	6/6 (100)	2/6 (33.3)	1/6 (16.7)	0/6 (0)	2/6 (33.3)	0/6 (0)
Bilateral grade 1	28/41 (68.3)	15/41 (36.6)	9/41 (22.0)	0/41 (0)	5/41 (12.2)	2/41 (4.9)
Unilateral grade 1 and contralateral grade 2	46/59 (78.0)	39/59 (66.1)	35/59 (59.3)	11/59 (18.6)	14/59 (23.7)	2/59 (3.4)
*P* value*	0.16	<0.001	<0.001	0.008	0.03	0.45

Values are *n*/*N* (%). *BME* bone marrow edema, *MRI* magnetic resonance imaging, *SIJ* sacroiliac joint, *SPARCC* Spondyloarthritis Research Consortium of Canada, *SSS* sacroiliac joint structural score

*Cochran-Mantel-Haenszel test *P* value for general association

A univariate analysis of the relationship between 23-DVU spinal score and select dichotomous patient characteristics, including structural lesions, determined that male patients and patients with SIJ BME had significantly higher 23-DVU spinal scores (see Additional file 1: Table S3). According to Spearman correlation, 23-DVU spinal score did not correlate with age, symptom duration, or individual structural lesions when evaluated as continuous risk factors (Spearman correlation –0.006 to 0.128). Multivariate stepwise regression analysis indicated that male sex and the presence of backfill are the best set of significant factors associated with 23-DVU spinal score; parameter estimates (SE): male sex: 2.8 (1.2), *P* = 0.02; backfill: 4.4 (1.7), *P* = 0.01.

Additional file 1: Table S4 presents the proportion of patients with individual lesion scores >0, according to two different definitions: at least one reader scored >0, and both readers scored >0. The table demonstrates that, as expected, the number of patients with structural lesions was somewhat higher using the first definition. The inter-observer ICC for structural lesions shows that, overall, there was little variation in the interpretations of the two readers (see Additional file 1: Table S5).

## Discussion

The results of this study demonstrate that structural lesions on MRI, particularly erosions, may occur in nr-axSpA when radiographs are normal or inconclusive, and even in the absence of SIJ BME on MRI. Additionally, mean 23-DVU spinal scores were higher in patients with SIJ structural lesions than without. On analysis of the patient demographics, in the group with SIJ BME, most patients with SIJ structural lesions were male and most were HLA-B27 positive. These data support the concept that nr-axSpA is an early stage of axSpA and that structural lesions in the SIJ are associated with a more severe phenotype characterized by more active spinal inflammation.

Prospective analysis will be necessary to determine whether assessment of these MRI structural lesions is of prognostic significance for the development of spinal ankylosis, which has much greater clinical significance than in the SIJ. Resolution of inflammation in erosions becomes evident on T1-weighted MRI scans by the appearance of a new lesion that fills the cavity in excavated bone [21]. This tissue is called “backfill” and is brighter than normal marrow on T1-weighted MRI. With further tissue remodeling, ankylosis may then develop at the site of backfill [9], at which point the backfill is no longer evident, as bright marrow traverses the joint space from the iliac to the sacral bones. Similar tissue that is brighter than normal marrow on T1-weighted MRI may be observed in subchondral bone marrow, especially following resolution of BME, and has been termed fat metaplasia [10]. It has been proposed that this tissue in subchondral bone and at erosions may reflect a reparative phenotype associated with an increased propensity toward ankylosis. A preliminary report suggests that the presence of fat metaplasia in the SIJ predicts development of spinal ankylosis [22], which would support this hypothesis.

In addition, the presence of structural lesions in the SIJ may reflect a more severe disease phenotype with progression to involve the spine, as recorded in our data by the higher 23-DVU spinal scores. It has been suggested that there is a window of opportunity for disease modification with anti-inflammatory agents by treating acute inflammatory lesions before bone formation pathways are triggered in more complex inflammatory lesions [23]. Consequently, the presence of structural lesions in the SIJ may help select patients for intervention with anti-TNF agents early in the disease course.

The analysis comparing SIJ BME and structural lesions with radiographic grade did not identify a linear relationship between these measurements, although the highest frequency of MRI structural lesions was observed in those cases with radiographic grade 1 and contralateral grade 2. MRI SIJ structural lesions occurred frequently in patients with normal SIJs on radiographs, reflecting the greater sensitivity of MRI over radiography for identification of structural lesions as well as inflammation.

There are several limitations to this study. One is the interpretation of the T1WSE MRI scans. The results of the inter-observer ICC analysis for the readings suggest that the scores provided by each reader were very similar. However, due to the complexities of scoring erosions and backfill on T1WSE MRI scans, it is necessary to use caution when interpreting the results. Erosion may be difficult to discern on T1WSE MRI because it requires loss of dark signal signifying a breach of cortical bone. Backfill is a heterogeneous lesion on T1WSE MRI, which requires both irregular dark signal signifying sclerotic bone together with bright signal medial to this signifying fat metaplasia. It is important to be clear that the study was not designed to draw conclusions on the diagnostic utility of structural lesions, because the population was selected for active disease and there were no controls. Further validation using computed tomography would be helpful for the understanding of both lesions.

## Conclusions

This study demonstrated that in patients with nr-axSpA, structural lesions, particularly erosions, may be present on MRI of the SIJ when radiographs are normal or inconclusive. Although structural lesions were more likely in patients with MRI SIJ BME, they were also present in the absence of MRI SIJ BME. Patients with structural lesions are more likely to be male, HLA-B27 positive, and have higher 23-DVU spinal scores. Prospective data are necessary to understand the prognostic implications associated with these lesions and the impact of treatment.

## Additional files

Additional file 1: **Table S1.** Pre-specified cases adjudicated by a blinded third reader. **Table S2.** SPARCC SSS for lesions, according to SIJ BME. **Table S3.** Univariate analysis of relationship between spinal inflammation and select patient characteristics at baseline. **Table S4.** Proportion of patients with SPARCC SSS lesions >0, according to two different definitions. **Table S5.** Inter-observer intra-class correlation coefficients for structural lesions. (PDF 100 kb)

Additional file 2: Lesions seen on T1 weighted spin echo MRI. (PDF 128 kb)

## Abbreviations

ASAS: Assessment of SpondyloArthritis international Society
axSpA: Axial spondyloarthritis
BASDAI: Bath Ankylosing Spondylitis Disease Activity Index
BASFI: Bath Ankylosing Spondylitis Functional Index
BASMI: Bath Ankylosing Spondylitis Metrology Index
BME: Bone marrow edema
BMI: Body mass index
CMH: Cochran-Mantel-Haenszel
DVU: Discovertebral unit
HLA: Human leukocyte antigen
IBD: Inflammatory bowel disease
ICC: Intra-class correlation coefficient
MRI: Magnetic resonance imaging
nr-axSpA: Non-radiographic axSpA
ns: Nonsignificant
NSAIDs: Nonsteroidal anti-inflammatory drugs
PsO: Psoriasis
SD: Standard deviation
SIJ: Sacroiliac joint
SPARCC: Spondyloarthritis Research Consortium of Canada
SSS: Sacroiliac joint structural score
STIR: Short tau inversion recovery
T1WSE: T1 weighted spin echo
TNF: Tumor necrosis factor
