# Supplementary material for: MRI evidence of structural changes in the sacroiliac joints of patients with non-radiographic axial spondyloarthritis even in the absence of MRI inflammation
Source: Arthritis Res Ther. 2017 Jun 6;19:126. doi: 10.1186/s13075-017-1342-9 (PMC5461761; doi:10.1186/s13075-017-1342-9)
Supplement: Supplementary file 1 — Table S1. Pre-specified cases adjudicated by a blinded third reader. Table S2. SPARCC SSS for lesions, according to SIJ BME. Table S3. Univariate analysis of relationship between spinal inflammation and select patient characteristics at baseline. Table S4. Proportion of patients with SPARCC SSS lesions >0, according to two different definitions. Table S5. Inter-observer intra-class correlation coefficients for structural lesions. (PDF 100 kb) [file 13075_2017_1342_MOESM1_ESM.pdf]

## Additional file 1

**Table S1.** Pre-specified cases adjudicated by a blinded third reader [1]

| **Case Description** |
| --- |
| • Cases read by one reader but considered unreadable by the second reader<br>• Cases in which readers' change scores differed in opposite directions (i.e., one positive, one negative) by ≥3 and ≥5 points for SPARCC sacroiliac joint and spinal scores, respectively<br>• Cases in which readers' change scores differed in the same direction (i.e., both positive or both negative) by ≥5 and ≥9 points for SPARCC sacroiliac joint and spinal scores, respectively* |

* The MRI pre and post sets were read by the adjudicator and the average of the adjudicator's score and the closest original score was used for analysis. Cutoffs for differences in SPARCC sacroiliac joint and spinal scores were determined by estimates of minimally important change [2]
*SPARCC* Spondyloarthritis Research Consortium of Canada

**Table S2.** SPARCC SSS for lesions, according to SIJ BME

| Structural lesion | SIJ BME | *N* | Mean (SD) | Median (Q1, Q3) | ANOVA *P* value | Wilcoxan-Mann-Whitney *P* value |
| --- | --- | --- | --- | --- | --- | --- |
| Erosion | ≥2 | 128 | 2.58 (3.45) | 1.0 (0, 4.0) | <0.001 | <0.001 |
| | <2 | 55 | 0.61 (1.69) | 0 (0. 0.5) | | |
| Backfill | ≥2 | 128 | 0.99 (2.37) | 0 (0, 0.5) | 0.004 | <0.001 |
| | <2 | 55 | 0.05 (0.17) | 0 (0, 0) | | |
| Fat | ≥2 | 128 | 0.50 (1.62) | 0 (0, 0) | 0.07 | 0.01 |
| metaplasia | <2 | 55 | 0.10 (0.57) | 0 (0, 0) | | |
| Ankylosis | ≥2 | 128 | 0.11 (0.82) | 0 (0, 0) | 0.60 | 0.76 |
| | <2 | 55 | 0.20 (1.42) | 0 (0, 0) | | |

*ANOVA* analysis of variance; *BME* bone marrow edema; *SIJ* sacroiliac joint; *SPARCC* Spondyloarthritis Research Consortium of Canada; *SSS* sacroiliac joint structural score

**Table S3.** Univariate analysis of relationship between spinal inflammation and select patient characteristics at baseline, including structural lesions

| | | | 23-DVU* spinal score | | |
|---|---|---|---|---|---|
| Baseline characteristic | Subgroup | *N* | Mean (SE) | Median (Q1, Q3) | *P* value† |
| Gender | Female | 73 | 2.4 (0.4) | 1.0 (0, 2.5) | |
| | Male | 110 | 5.8 (0.9) | 2.1 (0, 7.0) | 0.03 |
| Ankylosis | 0 | 179 | 4.4 (0.6) | 1.5 (0, 5.5) | |
| | >0 | 4 | 9.0 (6.7) | 3.8 (0.5, 17.5) | 0.59 |
| Erosion | 0 | 119 | 3.2 (0.4) | 1.5 (0, 4.0) | |
| | >0 | 64 | 6.8 (1.5) | 2.0 (0.3, 7.3) | 0.07 |
| Any lesion | 0 | 107 | 3.2 (0.5) | 1.5 (0, 4.0) | |
| | >0 | 76 | 6.3 (1.3) | 2.0 (0.3, 7.3) | 0.11 |
| Backfill | 0 | 157 | 3.7 (0.5) | 1.5 (0, 5.0) | |
| | >0 | 26 | 8.8 (3.0) | 2.3 (0.5, 12.0) | 0.25 |
| Fat metaplasia | 0 | 168 | 4.4 (0.6) | 1.5 (0, 5.0) | |
| | >0 | 15 | 5.5 (1.9) | 3.0 (0.5, 8.0) | 0.36 |
| SPARCC SIJ | <2 | 55 | 2.5 (0.5) | 1.0 (0, 3.5) | |
| BME | ≥2 | 128 | 5.3 (0.8) | 2.0 (0.3, 6.5) | 0.02 |

*Range, 0–414
†From Wilcoxon-Mann-Whitney test
*DVU* discovertebral units; *SIJ* sacroiliac joint; *SPARCC* Spondyloarthritis Research Consortium of Canada

**Table S4.** Proportion of patients with SPARCC SSS lesions >0, according to 2 different definitions

| Structural lesion | At least 1 reader >0<br>*N*=185<br>*n* (%) | Both readers >0<br>*N*=185<br>*n* (%) |
|---|---|---|
| Any lesion | 98 (53.0) | 77 (41.6) |
| Erosion | 91 (49.2) | 65 (35.1) |
| Backfill | 44 (23.8) | 26 (14.1) |
| Fat metaplasia | 24 (13.0) | 15 (8.1) |
| Ankylosis | 8 (4.3) | 4 (2.2) |

*SPARCC* Spondyloarthritis Research Consortium of Canada; *SSS* sacroiliac joint structural score

**Table S5.** Inter-observer intra-class correlation coefficients for structural lesions

| Structural lesion | ICC |
|---|---|
| Fat metaplasia | 0.875 |
| Erosion | 0.847 |
| Backfill | 0.776 |
| Ankylosis | 0.976 |

*ICC* intra-class correlation coefficients

## References


1. Dougados M, van der Heijde D, Sieper J, Braun J, Maksymowych WP, Citera G et al. Symptomatic Efficacy of Etanercept and Its Effects on Objective Signs of Inflammation in Early Nonradiographic Axial Spondyloarthritis: A Multicenter, Randomized, Double-Blind, Placebo-Controlled Trial. Arthritis Rheum. 2014;66:2091-102.

2. Maksymowych WP, Lambert RG, Brown LS, Pangan AL. Defining the Minimally Important Change for the SpondyloArthritis Research Consortium of Canada Spine and Sacroiliac Joint Magnetic Resonance Imaging Indices for Ankylosing Spondylitis. J Rheumatol. 2012;39:1666-74.
