# Supplementary material for: MRI evidence of structural changes in the sacroiliac joints of patients with non-radiographic axial spondyloarthritis even in the absence of MRI inflammation
Source: Arthritis Res Ther. 2017 Jun 6;19:126. doi: 10.1186/s13075-017-1342-9 (PMC5461761; doi:10.1186/s13075-017-1342-9)

Additional file 2

Lesions seen on T1 weighted spin echo MRI: subchondral fat metaplasia (**a**), backfill (**b**), erosion (**c**), and ankylosis (**d**)

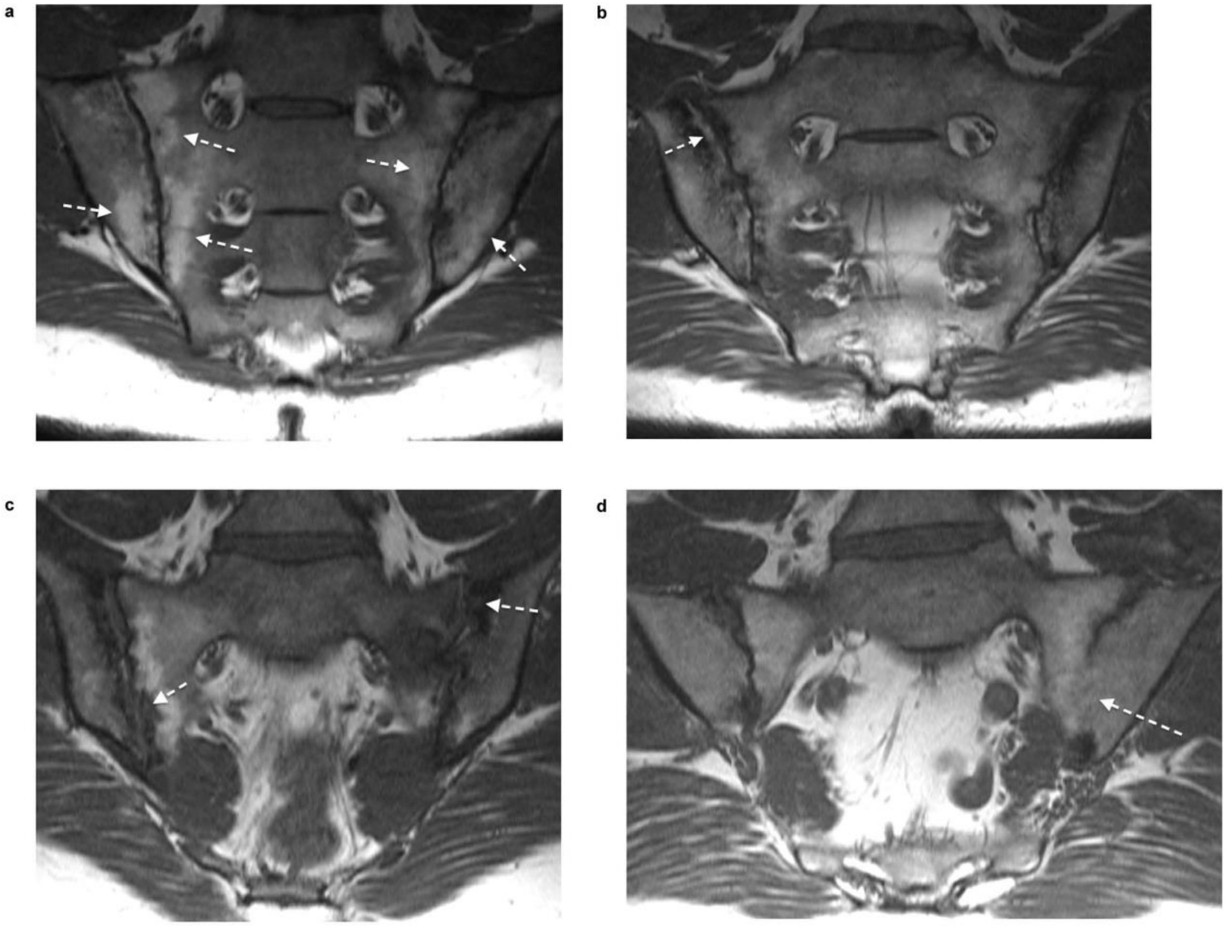

Supplement: Supplementary file 2 — Lesions seen on T1 weighted spin echo MRI. (PDF 128 kb) [file 13075_2017_1342_MOESM2_ESM.pdf]
